# Revolutionizing Early-Stage Cervical Cancer Treatment: A Comprehensive Review of Radical Trachelectomy as a Minimally Invasive Approach

**DOI:** 10.7759/cureus.53958

**Published:** 2024-02-10

**Authors:** Drashti Patel, Surekha Tayade, Aditi Singh Thakur, Sukanya Singh

**Affiliations:** 1 Obstetrics and Gynaecology, Jawaharlal Nehru Medical College, Datta Meghe Institute of Higher Education & Research, Wardha, IND

**Keywords:** oncological outcomes, fertility preservation, minimally invasive approach, early-stage cervical cancer, radical trachelectomy

## Abstract

This comprehensive review explores the transformative potential of radical trachelectomy as a minimally invasive approach to early-stage cervical cancer treatment. Cervical cancer, a significant global health concern, necessitates innovative strategies for effective intervention, particularly in its early stages. The review begins by providing a background on cervical cancer, emphasizing the pressing need for early-stage treatment options. The focal point is the meticulous examination of radical trachelectomy, a surgical technique that addresses the oncological aspects of treatment and preserves fertility. The conclusion encapsulates vital findings, highlighting this approach's dual benefits and challenges. Furthermore, the implications for clinical practice underscore the paradigm shift that radical trachelectomy brings, urging healthcare professionals to consider its integration into personalized treatment plans. The review concludes with a compelling call to action for further research, emphasizing the importance of refining surgical techniques and fostering interdisciplinary collaboration to ensure the seamless implementation of radical trachelectomy. Overall, this review sets the stage for a transformative shift in the approach to early-stage cervical cancer, presenting radical trachelectomy as a promising frontier in the quest for effective and patient-centered interventions.

## Introduction and background

Cervical cancer is predominantly caused by persistent infection with high-risk human papillomavirus (HPV) types, notably HPV 16 and 18. It arises from abnormal changes in the cells of the cervix, the lower part of the uterus connecting to the vagina. Despite preventive measures such as HPV vaccination and regular screening, cervical cancer continues to affect a substantial number of women, particularly in developing regions with limited access to healthcare resources [1]. The disease typically progresses slowly, allowing for early detection and intervention. However, without timely diagnosis and appropriate treatment, cervical cancer can advance to later stages, leading to higher morbidity and mortality rates. Understanding the nuances of early-stage treatment options becomes crucial in mitigating the impact of cervical cancer on women's lives [1].

Early detection of cervical cancer significantly improves the chances of successful treatment and long-term survival. Current standard treatment options for early-stage cervical cancer include surgery, radiation therapy, and chemotherapy. While these modalities have shown efficacy, they often have associated side effects and impact patients' quality of life. There is a pressing need for innovative, minimally invasive approaches that not only effectively treat cancer but also minimize the physical and emotional burden on patients [2].

Radical trachelectomy, emerging as a groundbreaking surgical technique, represents a paradigm shift in the management of early-stage cervical cancer. This minimally invasive procedure aims to remove the cancerous tissue while preserving the uterus, offering a unique balance between oncological outcomes and fertility preservation. As we embark on a detailed exploration of this approach, this review seeks to unravel the intricacies of Radical trachelectomy and its potential to redefine the landscape of cervical cancer treatment.

## Review

Radical trachelectomy: definition and historical perspective

Definition of Radical Trachelectomy

Radical trachelectomy is a surgical intervention characterized by the excision of the cervix, upper vaginal region, and adjacent tissues, all while retaining the integrity of the uterus. This meticulous procedure is designed to address early-stage cervical cancer with a particular emphasis on preserving the patient's fertility. Alternately termed radical cervicectomy or radical vaginal trachelectomy, this surgery can be executed through diverse methodologies, encompassing both open and minimally invasive techniques. Noteworthy for its safety and efficacy among eligible patients, extensive research has substantiated the procedure's favorable outcomes in oncology and obstetrics [3-5].

Evolution of the Procedure

The evolution of radical trachelectomy over the past decade marks a significant advancement in the realm of fertility-sparing surgeries for early-stage cervical cancer. Pioneered by several centers globally, this procedure has been refined and developed following groundbreaking work. Its execution involves various methods, encompassing both open and minimally invasive techniques. Particularly noteworthy is the emergence of minimally invasive radical trachelectomy as a viable alternative to open surgery, catering to patients with early-stage cervical cancer who wish to preserve fertility. In examining the historical context, the roots of radical hysterectomy, a related procedure, can be traced back to the late 19th century, undergoing several modifications and refinements over time [6,7].

Surgical Techniques and Variations

The evolution of radical trachelectomy has unfolded over the past decade, propelled by the pioneering efforts of several centers worldwide [6]. Intriguingly, this procedure, once nearly forgotten for four decades, experienced a renaissance through the endeavors of the teams led by Smith et al. and Ungár et al. in the early 2000s [8]. Rediscovered and revitalized, radical trachelectomy has since gained substantial popularity as a fertility-sparing approach for early-stage cervical cancer. Its resurgence underscores the dynamic nature of medical advancements and the potential for innovative solutions to reemerge and redefine clinical practices.

Vaginal radical trachelectomy: Vaginal radical trachelectomy stands as a sophisticated surgical approach involving the meticulous removal of both the cervix and upper vagina through a strategically placed vaginal incision. This method, marked by its precision and attention to anatomical nuances, epitomizes a minimally invasive technique. Notably, it distinguishes itself by leaving no discernible external scars, underscoring its cosmetic advantage and aligning to minimize postoperative morbidity [4].

Abdominal radical trachelectomy (ART): The intricacies of abdominal radical trachelectomy unfold in its comprehensive removal of the cervix, upper vagina, and adjacent tissues, orchestrated through a strategic abdominal incision. While the available published results of ART in direct comparison with its vaginal counterpart may be somewhat constrained, the existing body of research suggests promising outcomes. Specific studies indicate that ART is proficient in preserving fertility and yields commendable oncological results. This underscores its potential as a pivotal surgical intervention in the continuum of cervical cancer management, particularly in cases where abdominal access is deemed a more viable and effective route [4].

Laparoscopic radical trachelectomy: Laparoscopic radical trachelectomy emerges as a progressive and refined approach involving the removal of the cervix and adjacent tissues with the aid of minimally invasive techniques, including robotic-assisted surgery. This method, characterized by its technological sophistication, offers a shorter learning curve for practitioners. Furthermore, its superior oncological radicality, when juxtaposed with the combination of vaginal radical trachelectomy and laparoscopic pelvic lymphadenectomy, underscores its efficacy and precision. This underscores its role as an advanced surgical modality, emphasizing technological innovation and procedural finesse in pursuing optimal patient outcomes [4].

Patient selection criteria

Eligibility for Radical Trachelectomy

Tumor size and histology: Initially, eligibility was confined to patients with stage IB1 tumors of favorable histology and sizes less than 2 cm. However, ongoing research has explored the feasibility of expanding these criteria to encompass tumors ranging from 2 to 4 cm and histological variations such as squamous, adenocarcinoma, or adenosquamous types [5]. This reflects an evolving understanding of the procedure's applicability to broader cases.

Desire to maintain fertility: A pivotal criterion for considering radical trachelectomy is a woman's explicit desire to preserve fertility. The procedure uniquely allows candidates to retain their uterus, presenting a viable option for those who wish to become pregnant post-treatment [5,6]. This patient-centric approach acknowledges the significance of reproductive goals in the decision-making process.

Specific histology criteria: Studies have indicated that radical trachelectomy is a viable option for women with squamous, adenocarcinoma, or adenosquamous histologies, provided there is a strong desire to maintain fertility [5]. This underscores the personalized nature of treatment decisions, aligning them with the individual characteristics of the cancer and the patient's reproductive goals.

Preoperative assessment: A thorough preoperative evaluation is paramount, involving imaging modalities such as MRI or PET scans, confirmation of low-risk histology through pathology assessment, and reproductive endocrinology consultation. This comprehensive assessment aims to explore alternative fertility options, including surrogacy and ensures that the chosen intervention aligns with the patient's overall health and well-being [5].

Age and parity: While most patients undergoing radical trachelectomy are young, with a median age of 31 years and a majority being nulliparous at the time of the procedure, age and parity are not rigid exclusion criteria. Successful patient selection involves a nuanced understanding of each individual's unique circumstances. The procedure is ideally suited for women with early-stage cervical cancer who wish to preserve fertility and fulfill specific histology and tumor size criteria [5].

Factors Influencing Decision-Making 

Fertility information provision during consultations: The adequacy of information about fertility preservation provided during medical consultations is pivotal in shaping the decision-making process for women contemplating fertility preservation treatment. When this information is inadequately conveyed, it becomes a substantial barrier, hindering women from making well-informed choices about their reproductive health. Comprehensive and clear communication in this context is imperative to empower women with the knowledge necessary for navigating treatment decisions [7].

Patient-provider communication: The quality of communication between patients and healthcare providers emerges as a critical determinant in the decision-making process. A barrier arises when women feel unable to initiate conversations about fertility preservation with their healthcare providers. Effective patient-provider communication is not just a means of relaying information but a vital channel for addressing concerns, understanding individual preferences, and fostering a collaborative decision-making process, especially in the context of cancer treatment decisions [7].

Desire to maintain fertility: The profound desire to preserve fertility stands as a potent influencer in the decision-making process for women facing cancer. This strong inclination toward maintaining reproductive options may lead women to explore fertility preservation treatments like radical trachelectomy. Acknowledging and understanding this desire's emotional and personal dimensions is crucial for tailoring treatment plans that align with patients' holistic well-being [5].

Health system factors: In low- and middle-income countries, systemic factors within the health system substantially influence decision-making. Issues such as a poor flow of information, the lack of prioritization of preventive services, and inadequate provision of screening services contribute to challenges in cancer screening uptake. These challenges, in turn, have a cascading effect on the decision-making process by limiting access to critical information and impacting the overall approach to treatment [8].

Oncologic and fertility outcomes: The availability of information on the oncologic and fertility outcomes of specific treatments, particularly radical trachelectomy, becomes a pivotal factor in decision-making. Positive outcomes regarding effective cancer control and fertility preservation contribute significantly to the acceptance of such treatments. This emphasizes the importance of evidence-based information in guiding women through decision-making and instills confidence in the chosen treatment approach [5].

Comparative Analysis with Traditional Treatment Options

Minimally invasive radical trachelectomy vs. open radical trachelectomy: A groundbreaking multicenter randomized controlled trial is currently underway, aiming to compare the oncologic and fertility outcomes of patients undergoing minimally invasive radical trachelectomy and open radical trachelectomy [9]. Preliminary results from a multi-center research initiative in China have provided intriguing insights. The study indicated that patients treated with ART exhibited superior oncological outcomes yet faced less favorable fertility outcomes compared to those undergoing vaginal radical trachelectomy (VRT) [10]. This ongoing research sheds light on the potential nuances between surgical approaches and underscores the importance of balancing oncologic efficacy and fertility preservation.

Radical trachelectomy vs. radical hysterectomy: Radical hysterectomy (RH) traditionally stands as the gold standard surgical procedure for early-stage cervical cancer. However, radical trachelectomy presents a compelling alternative, offering the unique advantage of fertility preservation [9]. A comprehensive systematic review of published literature has provided encouraging insights, suggesting that radical trachelectomy demonstrates favorable surgical, oncologic, and pregnancy outcomes. Despite these promising findings, higher-level evidence is deemed essential to establish the efficacy of radical trachelectomy compared to the standard RH procedure [6]. This reflects the ongoing need for rigorous research to inform treatment decisions.

Fertility preservation: One of the primary merits of radical trachelectomy lies in its potential to preserve fertility among eligible patients. A comparative analysis comparing vaginal and abdominal radical trachelectomy for early-stage cervical cancer highlighted that VRT patients experienced more favorable fertility outcomes [10]. Radical trachelectomy, therefore, emerges as a valuable alternative for women with early-stage cervical cancer who wish to preserve their fertility. However, the optimal surgical approach and a comprehensive understanding of the comparative oncological and fertility outcomes between radical trachelectomy and traditional treatment options remain subjects that warrant further investigation. The evolving research landscape in this domain is poised to provide critical insights that can refine clinical decision-making and improve patient outcomes.

Benefits and challenges of radical trachelectomy

Minimally Invasive Nature

Minimally invasive radical trachelectomy has emerged as a promising alternative to open surgery for individuals with early-stage cervical cancer who seek to preserve fertility, offering a spectrum of benefits. Notably, this approach is associated with a swifter recovery period, diminished postoperative discomfort, and reduced scarring, making it an appealing choice for eligible patients [11,12]. The specific advantages of the vaginal approach are accentuated by its capacity for an even faster recovery and quicker return to daily activities. This feature significantly contributes to the patient's overall well-being [11]. The broader acceptance of minimally invasive surgery in this context is underscored by its perceived benefits, encompassing a reduced hospital stay and a decreased likelihood of adhesions, further enhancing the appeal of this approach [9]. However, it is crucial to acknowledge the challenges intrinsic to the minimally invasive approach. The vaginal approach, in particular, demands a specialized skill set in complex vaginal surgery, necessitating adequate training for practitioners to ensure proficiency and optimal patient outcomes [11]. Additionally, while the open approach may be perceived as more radical regarding parametrial and paracervical resection, it carries theoretical drawbacks that warrant careful consideration [9]. Striking the right balance between the advantages and challenges of minimally invasive radical trachelectomy becomes imperative, especially compared to the open surgical approach.

Preservation of Fertility

The preservation of fertility stands out as a paramount benefit of radical trachelectomy, a surgical intervention designed to address early-stage cervical cancer. This procedure, recognized as the sole approved fertility-sparing approach for young patients with early cervical cancer, involves the meticulous removal of the cervix, upper vagina, and surrounding tissues while retaining the uterus [13]. The distinctive advantage of radical trachelectomy lies in allowing patients to conceive and carry a pregnancy to term, marking a significant breakthrough in the realm of fertility preservation for those affected by cervical cancer [14]. Following conization or trachelectomy, physicians typically recommend a waiting period of 6 to 12 months before attempting to conceive, ensuring optimal recovery and reducing potential risks [14].

Radical trachelectomy can be performed using various methods, including open and minimally invasive techniques. The minimally invasive approach, mainly through the vaginal route, holds noteworthy appeal due to its associated faster recovery and quicker return to daily activities compared to open surgery [13]. Surgeons, whenever feasible, opt for minimally invasive radical trachelectomy, employing techniques such as laparoscopy or robotic surgery to enhance patient outcomes and experiences [15]. In addition to radical trachelectomy, another fertility-preserving option available to women with cervical cancer is oocyte cryopreservation, commonly known as freezing eggs [15]. This provides an alternative avenue for preserving fertility, complementing the benefits offered by radical trachelectomy.

Reduced Morbidity and Quality of Life

Radical trachelectomy, a surgical intervention designed to treat early-stage cervical cancer while preserving fertility, has demonstrated notable advantages in terms of reduced morbidity and improved quality of life. A comprehensive 2-year prospective study comparing the emotional, sexual, and overall quality of life concerns of women undergoing radical trachelectomy against those undergoing adjuvant radiotherapy revealed that radical trachelectomy was associated with superior quality of life outcomes [16]. This signifies a pivotal advancement in patients' holistic care, emphasizing oncological efficacy and the overall well-being of individuals undergoing this innovative procedure. Furthermore, studies exploring specific techniques, such as robot-assisted radical trachelectomy, have reported favorable outcomes, including low recurrence rates, high fertility rates, and successful term pregnancies [17]. This underscores the potential of advanced surgical approaches to enhance oncological and reproductive outcomes for patients undergoing radical trachelectomy.

However, it is essential to acknowledge that radical trachelectomy, despite its benefits, may present certain postoperative and long-term complications, including cervical stenosis, urogynecological morbidity, and lymphedema [18]. The choice of surgical approach adds a layer of consideration, with the vaginal approach offering faster recovery and a quicker return to daily activities. Nevertheless, this method requires specialized training in complex vaginal surgery [11]. Notably, despite the potential for complications, the overall morbidity associated with radical trachelectomy remains comparable to other surgical approaches, such as radical abdominal or radical vaginal hysterectomy. At the same time, recurrence rates exhibit similar levels [19].

Oncological Outcomes and Recurrence Rates

Research findings consistently highlight that radical trachelectomy, a surgical strategy employed to address early-stage cervical cancer while preserving fertility, boasts comparable oncological outcomes and recurrence rates when juxtaposed with other surgical approaches for early-stage cervical cancer. A systematic review scrutinizing laparoscopic and abdominal radical hysterectomy revealed strikingly similar 5-year recurrence-free survival rates between the two procedures, underscoring the efficacy of radical trachelectomy [20]. The extensive International Radical Trachelectomy Assessment Study corroborated these findings, reporting no discernible difference in recurrence rates at 4.5 years between open and minimally invasive surgery [21]. A study delving into the oncological and reproductive feasibility of abdominal radical trachelectomy further supported the credibility of this approach, reporting recurrence rates of less than 6% and mortality rates of less than 3% [22]. Comparisons between radical trachelectomy and other established surgical methods, such as radical abdominal or radical vaginal hysterectomy, have consistently indicated similar morbidity and recurrence rates, validating radical trachelectomy as a viable and effective treatment option for early-stage cervical cancer [19]. The wealth of evidence from these studies collectively affirms that radical trachelectomy is on par with other surgical approaches concerning oncological outcomes and recurrence rates.

Complications and Considerations

Radical trachelectomy, a surgical intervention employed for early-stage cervical cancer and designed to preserve fertility, comes with a spectrum of potential complications and considerations that warrant careful acknowledgment and communication with patients [23]. Intraoperative complications are a crucial facet of this surgical procedure, encompassing risks such as vascular trauma, ureteral injury, and harm to other intra-abdominal structures. Furthermore, the postoperative phase may introduce complications like urinary tract infection, lymphocele, vulvar edema, vulvar hematoma, and lymphedema, among others [23]. Patients must be apprised of these potential short- or long-term obstetrical, gynecological, and fertility complications, emphasizing the importance of informed decision-making.

Regarding the recovery process, the vaginal approach to radical trachelectomy holds the advantage of facilitating a faster return to daily activities. However, this approach necessitates specialized training in complex vaginal surgery [11]. While the potential for complications exists, robot-assisted radical trachelectomy has shown promising outcomes with a low recurrence rate and a high fertility rate [17]. This highlights the importance of considering various surgical techniques and their associated outcomes when discussing treatment options with patients.

Despite its merits, patients should be informed about potential challenges and considerations associated with radical trachelectomy. Importantly, discussions should encompass the possibility of abandoning the procedure in some instances and the prospect that final pathology may reveal a higher risk for recurrence, necessitating additional treatments [23]. This comprehensive approach to patient education ensures that individuals considering radical trachelectomy for fertility preservation in early-stage cervical cancer are equipped with a thorough understanding of both the potential benefits and associated complications. Informed decision-making, based on transparent communication about potential risks and outcomes, is integral to ensuring patient autonomy and well-being throughout the treatment process.

Surgical innovations and technological advances

Robotic-Assisted Radical Trachelectomy

Robotic-assisted radical trachelectomy stands as a cutting-edge and minimally invasive surgical procedure in gynecologic oncology, specifically designed for the treatment of early-stage cervical cancer. Leveraging high-definition 3D magnification, robotic technology, and miniature instruments, this advanced approach enables the precise removal of the cervix and a small amount of surrounding tissue. Incorporating robotic technology provides several notable advantages, including heightened precision, an enhanced range of movement, and a reduced risk of injury to delicate nerves and blood vessels. These features collectively contribute to the safety and efficacy of the procedure, positioning robotic-assisted radical trachelectomy as a significant advancement in the field [24,25].

Numerous studies have substantiated robotic technology's safety, feasibility, and improved surgical outcomes in gynecologic oncology, particularly in radical trachelectomy [12,24]. The procedure's applicability extends to the treatment of early cervical cancer, showcasing its potential to preserve fertility while offering the benefits of minimally invasive techniques. This innovation in surgical technology aligns with evolving trends in gynecologic oncology and signifies a paradigm shift towards enhancing patient outcomes through advanced surgical approaches.

Robotic-assisted radical trachelectomy, an amalgamation of precision and minimally invasive capabilities, represents a significant stride in gynecologic oncology. Beyond its technical prowess, the procedure holds the promise of improving patient outcomes, especially in terms of fertility preservation, and underscores the potential for expanding the scope of minimally invasive procedures in the realm of early-stage cervical cancer treatment [12,24]. This surgical innovation reflects the dynamic intersection of technology and medical practice, showcasing how advancements in robotic surgery can significantly contribute to the evolution of treatment options and outcomes for patients with gynecologic malignancies.

Image-Guided Navigation

Image-guided surgical navigation, or computer-assisted surgery, has emerged as a transformative technology with significant applications across various surgical procedures, ranging from endoscopic and neurosurgical interventions to tumor surgery. This cutting-edge technology integrates improved optics, advanced lighting systems, and state-of-the-art imaging capabilities to generate a precise replica of the target anatomical structure. The result is an invaluable tool that empowers surgeons to plan and execute procedures with enhanced accuracy and safety [26]. In its evolution, image-guided surgical navigation has played a pivotal role in advancing robotic technologies. It has become a cornerstone in surgical decision-making, particularly in neurosurgery, stereotaxy, and medical imaging [26,27]. This technology's ability to provide real-time, three-dimensional visualization of anatomical structures has revolutionized how surgeries are approached, ensuring higher precision and safety.

The impact of image-guided navigation is particularly noteworthy in complex endoscopic endonasal surgery. This technology has not only improved standard practices but has also introduced significant advantages in precision and safety, setting new benchmarks for the field [28]. Additionally, its utilization in tumor surgery demonstrates a seamless integration of frameless stereotactic localization technology with real-time video processing. This integration allows surgical navigation assistance without constraining the physician's judgment, providing a dynamic tool that enhances decision-making based on virtual computer-generated anatomical structures and real-time observations [29]. In essence, image-guided surgical navigation represents a substantial technological leap forward, significantly enhancing the precision and safety of various surgical interventions. Its integration into modern surgical practice has become essential, offering surgeons an invaluable resource to navigate complex anatomical structures confidently and accurately. As technology advances, the role of image-guided navigation will likely expand, further shaping the landscape of surgical innovation and patient care.

Future Prospects and Emerging Technologies

Robotics: Robot-assisted surgery has rapidly evolved, ushering in a new era of surgical precision, accuracy, and dexterity. Integrating robotic technology into surgical procedures holds immense potential to standardize surgical techniques, enhancing overall patient care. One of its notable advantages lies in its capacity to potentially reduce the learning curve for surgeons, making complex procedures more accessible and reproducible [30]. As a transformative force in the field, robotics not only refines surgical techniques but also opens avenues for innovation that can positively impact surgical outcomes and patient experiences.

3D printing: 3D printing in surgery represents a groundbreaking frontier, enabling the creation of customized solid objects from digital files. This technology has found applications across various surgical specialties, offering a range of benefits. Notably, it facilitates more minimally invasive surgery by providing surgeons with precise anatomical models for preoperative planning. This, in turn, contributes to improved patient outcomes, demonstrating the potential of 3D printing to revolutionize surgical approaches and enhance the overall efficacy of interventions [30]. Creating patient-specific models allows for a level of customization and precision that was previously unparalleled, marking a significant advancement in surgical technology.

Optical imaging and image-guided surgical navigation: High-tech advances in optical imaging and image-guided surgical navigation have ushered in an era of greater precision and less invasive surgical techniques. These technologies enable surgeons to visualize internal structures with enhanced clarity, improving their ability to navigate complex anatomies. Integrating image-guided navigation optimizes surgical outcomes, allowing for more targeted and efficient procedures. This contributes to the overall success of surgeries and minimizes the invasiveness of specific interventions, potentially leading to faster recovery times and reduced postoperative complications [31]. The synergy between optical imaging and navigation systems exemplifies how technological advancements can synergize to elevate surgical precision and patient care.

Collaboration and ethical considerations: The ongoing evolution of surgical technology necessitates collaboration between technology developers, healthcare institutions, and regulatory bodies. Building a conducive environment for innovation involves fostering partnerships that ensure the seamless integration of technological advancements into clinical practice. Simultaneously, ethical considerations play a pivotal role as technology continues to advance. Striking a balance between innovation and ethical standards is crucial to ensure patient safety, privacy, and overall well-being. A thoughtful and collaborative approach is essential to navigate the ethical implications of emerging technologies in surgery [32]. By establishing a framework that encourages responsible innovation, the surgical community can harness the full potential of technological advancements while safeguarding the interests of patients and upholding ethical standards.

Comparative analysis with alternative treatments

Radical Hysterectomy

Numerous studies have undertaken a comprehensive examination of outcomes associated with RH compared to alternative treatments for early-stage cervical cancer, yielding insightful findings that contribute to the nuanced understanding of therapeutic options. In a multicentric study conducted in France, an exploration of laparoscopic and abdominal radical hysterectomy revealed no discernible difference in survival rates for individuals with early-stage cervical cancer [33]. Building on this, a meta-analysis highlighted that laparoscopic radical hysterectomy (LRH) not only demonstrated faster functional recovery but also showcased reduced estimated blood loss when compared to the conventional open radical hysterectomy (ORH) [34]. These findings underscore the potential advantages of laparoscopic approaches regarding patient recovery and perioperative outcomes. Beyond traditional radical hysterectomy, minimally invasive radical trachelectomy has emerged as a noteworthy alternative for patients with early-stage cervical cancer who seek fertility preservation [12]. This technique offers a distinctive option, allowing for the removal of the cervix and upper vagina while preserving the uterus, catering to the specific reproductive goals of eligible patients.

Exploring alternative treatments, a study comparing oncologic outcomes between radical hysterectomy and primary concurrent chemoradiotherapy in women with bulky IB and IIA cervical cancer revealed that RH provided superior locoregional control and relapse-free survival in the high-risk group [35]. Moreover, a comparative analysis between radical hysterectomy followed by tailored adjuvant therapy and primary chemoradiation therapy in IB2 and IIA2 cervical cancer suggested that patients undergoing RH followed by tailored adjuvant therapy experienced enhanced survival outcomes in comparison to primary chemoradiation therapy [36]. These findings contribute valuable insights into the nuanced decision-making process surrounding treatment modalities for early-stage cervical cancer, particularly in high-risk scenarios.

Chemoradiation Therapy

A comprehensive comparative analysis has been undertaken to assess the efficacy of RH followed by tailored adjuvant therapy in contrast to primary chemoradiation therapy (CRT) for patients with early-stage cervical cancer. Recent studies have consistently indicated that patients who underwent RH followed by tailored adjuvant therapy experienced superior survival outcomes compared to those undergoing primary CRT [36]. This finding underscores the potential advantages of a surgical approach followed by personalized adjuvant interventions in optimizing patient outcomes. In a separate study focusing on locoregional control and relapse-free survival, RH demonstrated notable benefits in the high-risk group compared to primary concurrent chemoradiotherapy [35]. This suggests that, particularly in cases where patients face heightened risks, a surgical approach with subsequent tailored adjuvant therapy may provide superior oncologic outcomes.

Addressing the surgical aspect, a French multicentric study explored the survival differences between minimally invasive surgery and laparotomy in patients treated with radical hysterectomy [33]. Notably, the study found no evidence of a disparity in survival outcomes between these two surgical approaches, underscoring the comparable efficacy of minimally invasive surgery in radical hysterectomy. Furthermore, in the realm of fertility-sparing surgery for early-stage cervical cancer, minimally invasive radical trachelectomy has emerged as a noteworthy alternative to open radical hysterectomy [9,12]. This is particularly relevant for patients desiring fertility preservation, as minimally invasive techniques offer advantages such as faster recovery and reduced postoperative discomfort.

Immunotherapy and Targeted Therapies

Radical hysterectomy: RH is a prevalent treatment modality for early-stage cervical cancer, involving the comprehensive removal of the uterus, cervix, and occasionally the ovaries. This surgical approach may be complemented by tailored adjuvant therapy, incorporating chemotherapy, radiation therapy, or a combination of both. Existing studies suggest that patients undergoing RH, followed by tailored adjuvant therapy, may experience enhanced survival outcomes compared to those receiving primary chemoradiation therapy [36]. However, uncertainties persist regarding the optimal treatment strategy, particularly for patients with bulky early-stage cervical cancer, necessitating further research for definitive conclusions.

Primary concurrent chemoradiotherapy: Primary concurrent chemoradiotherapy is a comprehensive treatment approach involving simultaneous administration of chemotherapy and radiation therapy, either preceding or succeeding surgery. Some studies have indicated comparable oncologic outcomes between RH and primary concurrent chemoradiotherapy, particularly within the high-risk group [35]. However, determining the optimal treatment strategy for patients with bulky tumors requires additional research to guide clinical decision-making.

Immunotherapy and targeted therapies: Immunotherapy and targeted therapies may be considered for patients facing recurrent or advanced disease. However, they are not standard for primary or adjuvant treatment in early-stage cervical cancer [37]. Immunotherapeutic interventions, such as programmed cell death protein 1 (PD-1) inhibitors, have shown promise in treating advanced cervical cancer. Conversely, targeted therapies focusing on entities like the epidermal growth factor receptor 2 (EGFR2) have not substantially improved overall survival. The application of these advanced treatments underscores their potential utility in specific cases, especially in the context of recurrent or advanced disease.

## Conclusions

In conclusion, this comprehensive review has illuminated the potential of radical trachelectomy as a groundbreaking and minimally invasive approach to early-stage cervical cancer treatment. The examination of this surgical technique has emphasized its capacity to revolutionize clinical practice by effectively addressing the dual goals of cancer control and fertility preservation. The recapitulation of key findings underscores the promising outcomes and challenges associated with radical trachelectomy, encouraging a nuanced understanding of its role in the therapeutic landscape. Notably, the implications for clinical practice suggest a paradigm shift, urging healthcare professionals to consider this innovative approach when tailoring treatment plans for women with early-stage cervical cancer. Moreover, the call to action resonates with the need for continued research, refinement of surgical techniques, and collaborative efforts to integrate radical trachelectomy into routine clinical care seamlessly. As we navigate the intersection of efficacy and fertility preservation, the evolving landscape of cervical cancer treatment stands poised for positive transformation, with radical trachelectomy at its forefront.
